# Predicting driving decline and assessing crash risk in a globally aging population

**DOI:** 10.1590/0004-282X-ANP-2022-E001

**Published:** 2022-02-28

**Authors:** Ganesh M. Babulal

**Affiliations:** 1 Department of Neurology, Washington University School of Medicine, St. Louis, MO, USA, Department of Neurology Washington University School of Medicine St. Louis MO USA; 2 Department of Psychology, Faculty of Humanities, University of Johannesburg, Johannesburg, South Africa Department of Psychology Faculty of Humanities University of Johannesburg Johannesburg South Africa; 3 Department of Clinical Research and Leadership, The George Washington University School of Medicine and Health Sciences, Washington, DC, USA Department of Clinical Research and Leadership The George Washington University School of Medicine and Health Sciences Washington DC USA

With the growth of the global population (7.920 billion, Jan 2022), the number of older adults (age 65 and older) will also increase when it reaches 1.5 billion by 2050. There is a parallel increase in life expectancy across many countries in the rapidly expanding older adult age stratum. Older adults are aging in place/independent residence, remaining in the workforce longer, and actively participating in their communities. In a similar vein, older drivers are one of the fastest-growing groups, representing a significant proportion of all drivers. In the United States, older adults age ≥ 65 will constitute 25% of all drivers by 2050^1^. In Europe and Japan, older drivers will make up a quarter of all drivers much earlier (2030)^2^. In Brazil, estimates of older drivers follow similar trends and range from 22-35%^3^. Conversely, motor vehicle crashes kill 1.35 million persons annually, are the eighth leading cause of death across all ages, cost $1.8 trillion (2010 USD) annually, and differentially impact low and middle income countries financially, along with a higher death rate^4^. Older adults are at the highest risk for injuries and mortality as a result of physiological decline and fragility. 

Driving is a multifaceted and dynamic activity that demands instantaneous, sustained, and synchronized deployment of sen sorimotor, cognitive, and affective systems. The coordination across multisystemic functions occurs in response to a rapidly changing environment with numerous stimuli (e.g., weather, road conditions, light, other drivers) while coping with internal conditions like stress, fatigue, and sleepiness. Despite driving being an overlearned task, age-related decline may moderately impact abilities (e.g., reaction time, strength, range-of-motion, sensation) that support the vehicle-driver task interface. While these modest and gradual changes may limit driving performance, they do not impact driving safety. However, chronic conditions like arthritis, cerebrovascular disease, neuropathy, cataracts, and dementia impair and increase the risk of crashes, related injury, and mortality among older drivers. As a result, significant research efforts aim to assess driving decline and identify risk factors among healthy and cognitively-normal (CN) older drivers.

Vasques and colleagues used an asymptomatic^5^, healthy cadre of older adults to determine whether common neuropsychological assessments were associated with high-risk driving conditions on a driving simulator. The simulator scenarios included navigating a complex intersection, overtaking another vehicle, navigating in rain, and dealing with a vehicle malfunction. Older drivers identified as high-risk exhibited more errors (or penalties) on the intersection, overtaking, and rain performance scenarios that confirm and validate the cognitive demand of those tasks. The Rey Auditory-Verbal Learning Test (RAVLT) was able to distinguish between older drivers, and scores were significantly lower among those classified as high risk compared to those at normal risk. The RAVLT assesses attention, learning, and short-term memory (encoding, storage, retrieval). Since the simulator scenarios were novel and not experienced before, the participants had to learn and react to the new task with minimal practice. As expected, the inverse correlation between low RAVLT scores and higher errors/penalties on the simulator tasks corroborate the innate learning, memory, and attentional skills required of both tasks. Unsurprisingly, general cognitive screens like the Mini-Mental State Examination (MMSE) and Addenbrooke's Cognitive Evaluation (ACE) were not associated with any group differences.

For decades, the search has endured for a single or multidomain neuropsychological test or a composite score that may forecast or provide prescient knowledge of crash risk or driving decline. Measures of visuomotor, attentional control, and/or executive function (e.g., Trail Making Test) can predict an increased likelihood of crash risk among those with mild cognitive impairment and prodromal dementia^6^. However, this pattern does not hold for cognitively-normal older adults. In studies of CN older adults and Alzheimer’s disease biomarkers, those with more abnormal biomarker levels made two-and-a-half more errors and were faster to fail a road test compared to those with normal biomarkers^7^^,^^8^. However, there no differences between independent neuropsychological measures or a composite z-score between the groups. The null findings with cognitive tasks were also found in a longitudinal study of naturalistic daily driving behavior^9^ and a study of depression and driving performance^10^. General screenings like the MMSE or ACE were never designed to assess the hierarchical complexity of driving. Even multidomain measurements like the RAVLT may not capture the cognitive load and sensorimotor process integration that driving demands.

 Assessment of driving decline among older adults is nuanced and can be conducted across controlled platforms like road tests and driving simulators or in daily driving via GPS dataloggers. Multimorbidity, medications, and the prescribing cascade may further obscure the sensitivity and specificity of assessing impairments. While general screens like the MMSE may not be clinically informative, multidomain measurements like the RAVLT, as shown by Vasques and colleagues, can provide insight into process-specific impairments. As a result, associations between these impairments and performance on a driving simulator may provide data on the likelihood of safe driving. Other approaches may include a combination of different risk factors like demographics (e.g., age), a composite z-score, biomarkers, self-report, and objective driving behavior to predict status and trajectory of driving decline. Driving performance (simulator, road test) and driving behavior (naturalistic) are complex sets of cognitive processes that topographically overlap, interact, and rapidly shift in response to internal and external stimuli as an individual navigates a route or trip. Assessment should be comprehensive and encompass subjective, objective, and collateral metrics of driving behavior, multidomain assessment of cognitive functioning, and the older driver’s expectations about and motivation to drive^11^. 
